# Davidone C Induces the Death of Hepatocellular Carcinoma Cells by Promoting Apoptosis and Autophagy

**DOI:** 10.3390/molecules26175219

**Published:** 2021-08-28

**Authors:** Ping Song, Huiqi Huang, Yuanren Ma, Chaoqun Wu, Xinzhou Yang, Ho-Young Choi

**Affiliations:** 1 School of Chemistry and Chemical Engineering, Qinghai University for Nationalities, Xining 810007, China; spzhe@126.com; 2 School of Pharmaceutical Sciences, South-Central University for Nationalities, Wuhan 430074, China; Hhuiqi@hotmail.com (H.H.); 13007136998@163.com (Y.M.); wcqscuec@126.com (C.W.); 3 College of Korean Medicine, Kyung Hee University, Seoul 02447, Korea

**Keywords:** davidone C, *Sophora davidii* (Franch.) skeels, anticancer, hepatocellular carcinoma, apoptosis, autophagy

## Abstract

Davidone C is a newly discovered flavonoid compound purified from the ethyl acetate-soluble fraction of *Sophora davidii* (Franch.) Skeels. This study explored the anti-tumor activity of davidone C on hepatocellular carcinoma HepG2 and Bel-7402 cells and its mechanism through MTT method, morphological observation, flow cytometry and Western blotting. The results showed that davidone C significantly inhibited the proliferation of HepG2 and Bel-7402 cells in a time- and dose-dependent manner. The morphological changes of apoptotic cells can be observed under an inverted microscope, such as cell floating, chromosome condensation, apoptotic bodies, and other phenomena. The expressions of Bax, cleaved caspase-9, cleaved caspase-3 and cleaved PARP increased with the increase of dosage while Bcl-2 decreased, suggesting that the apoptotic mechanism might be related to the mitochondrial apoptotic pathway. Moreover, davidone C administration can down-regulate the expression of Grp78, and simultaneously up-regulate the expression of caspase-7 and caspase-12, indicating that the apoptotic mechanism might be related to the ERS pathway. In addition, davidone C can down-regulate the expression of p62, and simultaneously up-regulate the expression of LC3-I and LC3-II with a quantitative dependence, suggesting that the mechanism of apoptosis may be related to the autophagy signal pathway. All these results showed davidone C has potential effects on hepatocellular carcinoma.

## 1. Introduction

Hepatocellular carcinoma was the most common primary liver cancer in clinical practice in the world [1], and it is thus an important medical problem. The morbidity and mortality of liver cancer are at the forefront of cancer types, and its survival rate is very low (18%). There will be approximately 906,000 new cases and 830,000 deaths in 2020 globally [2]. Liver cancer is listed as the sixth most common tumor and the third leading cause of death from cancer [3]. The main risk factors include infection with hepatitis B or C virus, and alcohol-related cirrhosis. Hepatocellular carcinoma has been the main cause of death in patients with liver cirrhosis, and its incidence is expected to increase in the future. Therefore, liver cancer poses a serious threat to human health. However, there are rarely reported clinical treatments for hepatocellular carcinoma, such as local surgery (ablation, chemotherapy/radioembolization), and the treatment of advanced patients with drugs such as sorafenib, which face poor patient selectivity and prone to drug resistance disadvantages such as toxic and side effects [4]. In order to further improve the survival rate of patients with hepatocellular carcinoma and improve treatment methods, it has become a top priority to find and discover new anti-cancer drugs.

Among different treatment strategies, chemotherapy was the most reliable choice for the treatment of liver cancer. Currently, more than 60% of anti-cancer drugs come from plants. For thousands of years, natural products have been the main source of medicines and have made great contributions to human health [5,6,7]. The drugs found in natural products such as paclitaxel and vinblastine have excellent therapeutic effects on tumors, but their therapeutic mechanisms have not been fully explored [8]. Traditional medicinal materials from many ethnic regions have been proven to have good anti-tumor effects in clinical practice [9]. In recent years, great progress has been made in the anti-cancer effects of certain monomer compounds, including apigenin, resveratrol, curcumin, berberine, etc. [10]. There have been related research reports on tumor microenvironment and immune regulation. When these drugs are used in combination with clinical chemotherapeutics, they have multiple effects, such as prolonging the survival time of patients, reducing the dose of chemotherapeutic drugs, reversing drug resistance, and reducing the adverse reactions of patients [11]. Studies have shown that the combination of vincristine, curcumin and cisplatin can enhance the sensitivity of tumor cells to cisplatin and reverse the resistance of cisplatin. The combination of *Tripterygium* *wilfordii* and cisplatin can enhance tumor cell apoptosis. Ginsenoside Rg3 enhancing the efficacy of paclitaxel are examples of very significant effects [12,13,14]. Therefore, it is feasible to find new compounds from natural resources to combat hepatocellular carcinoma, which can meet the growing demand for the development of chemotherapy.

In this study, we obtained davidone C, which is a new flavonoid compound, isolated from ethyl acetate soluble fraction of *Sophora davidii* (Franch.) Skeels by comprehensive column chromatography, high performance liquid chromatography (HPLC) and other separation and purification methods. We conducted a preliminary screening of its cytotoxic activity and found that it showed good cytotoxicity to two liver cell lines HepG2 and Bel-7402. Therefore, in the current study, we evaluated the hepatotoxicity of Davidone C, and initially clarified the anti-proliferative mechanism of davidone C in HepG2 and Bel-7402 cells.

## 2. Results

### 2.1. Effects of Davidone C on Cell Viability in HepG2 and Bel-7402 Cells

**Figure 1:**
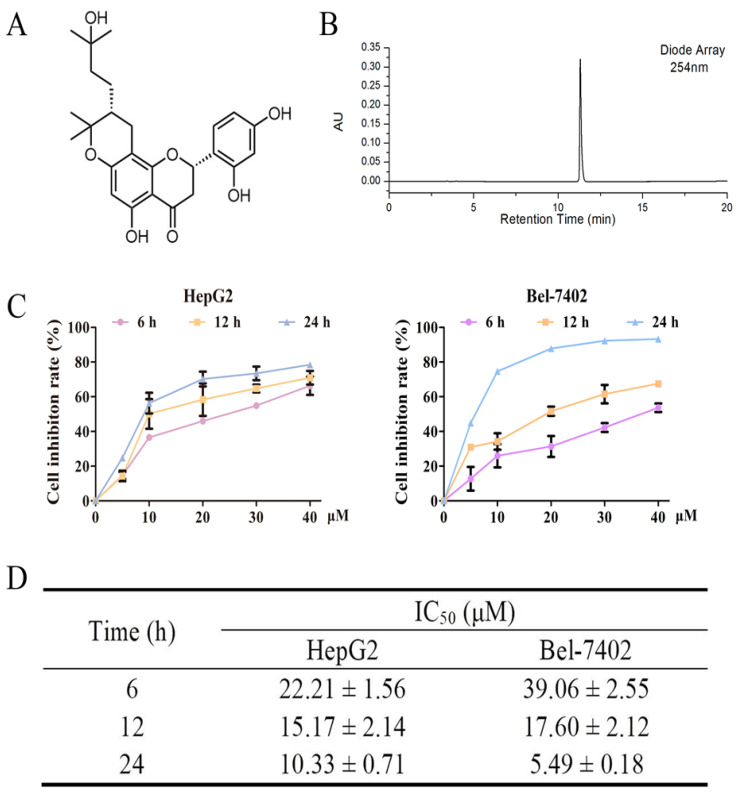
Davidone C-induced apoptosis of human hepatocellular carcinoma cells. (**A**) The chemical structure of davidone C; (**B**) The HPLC chromatogram of davidone C at 254 nm; (**C**) The cell inhibition rate of HepG2 and Bel-7402 cells treated with the specified concentration of Davidone C; (**D**) The IC_50_ values of HepG2 and Bel-7402 cells at 6, 12 and 24 h.

The compound davidone C was isolated and identified from the ethyl acetate-soluble part of *S. davidii*. Figure 1A,B show the chemical structure and a HPLC chromatogram of davidone C. The purity of davidone C was more than 95% based on the HPLC analysis.

We used the MTT method to study the anti-proliferative effects of davidone C in HepG2 and Bel-7402 cells (Figure 1C,D). It was observed that davidone C had a good inhibitory effect on HepG2 cells at 12 h (IC_50_ = 15.17 µM) and 24 h (IC_50_ = 10.33 µM) than 6 h. Davidone C also showed a good inhibitory effect on Bel-7402 cells at 12 h (IC_50_ = 17.60 µM) and 24 h (IC_50_ = 5.49 µM) than 6 h. In addition, the dose- and time-dependent chart showed that davidone C inhibits the growth of human liver cancer cell lines in a dose- and time-dependent manner.

### 2.2. Effects of Davidone C on Cell Migration and Related Protein Expression in HepG2 and Bel-7402 Cells

**Figure 2:**
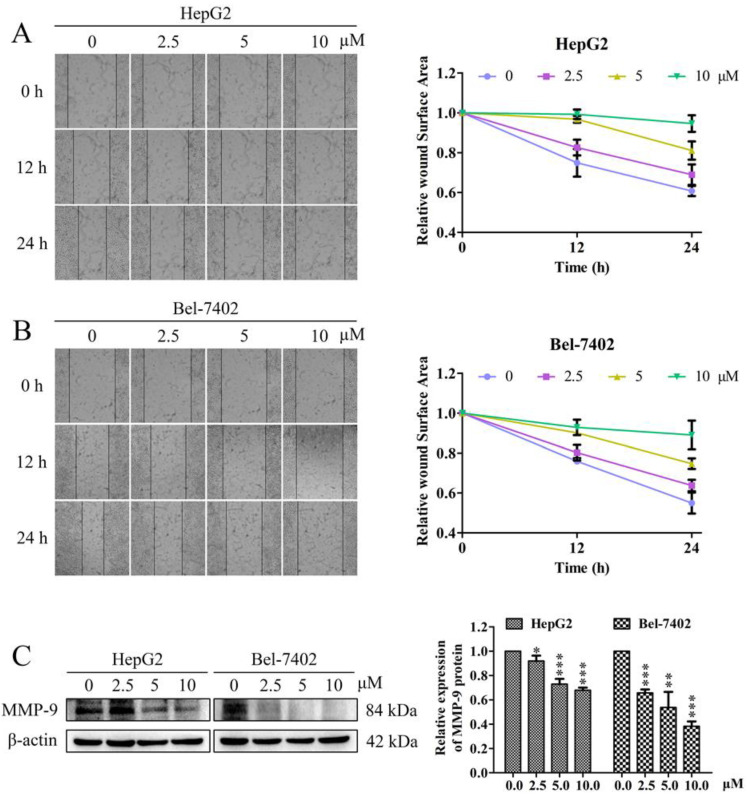
The inhibitory effect of davidone C on cell migration in HepG2 and Bel-7402 cells. (**A**,**B**) Representative images of scratch determination and counting results; (**C**) Western blot detection of MMP-9 protein expression results (* *p* < 0.05, ** *p* < 0.01 or *** *p* < 0.001).

Cell migration is a sign of cancer invasion. The cell scratch test is an effective method to evaluate the migration ability of cancer cells. In our study, the effect of davidone C on the migration of HepG2 and Bel-7402 cells was determined by cell scratch experiment. The scratch test (Figure 2A,B) showed that in HepG2 and Bel-7402 cells, davidone C (2.5, 5, 10 µM) significantly reduced cell migration after 12 h and 24 h. The relative wound surface areas of 5 and 10 µM groups were more than 0.7 at 12 h and more than 0.6 at 24 h, while the inhibitions on HepG2 and Bel-7402 cells proliferation at those concentrations were inconspicuous.

In addition, we performed Western blot detection on the expression of the key protein MMP-9 related to cell migration. The results showed that davidone C down-regulated the expression of MMP-9 (Figure 2C).

### 2.3. Effects of Davidone C on Morphological Changes and Apoptosis in HepG2 and Bel-7402 Cells

**Figure 3:**
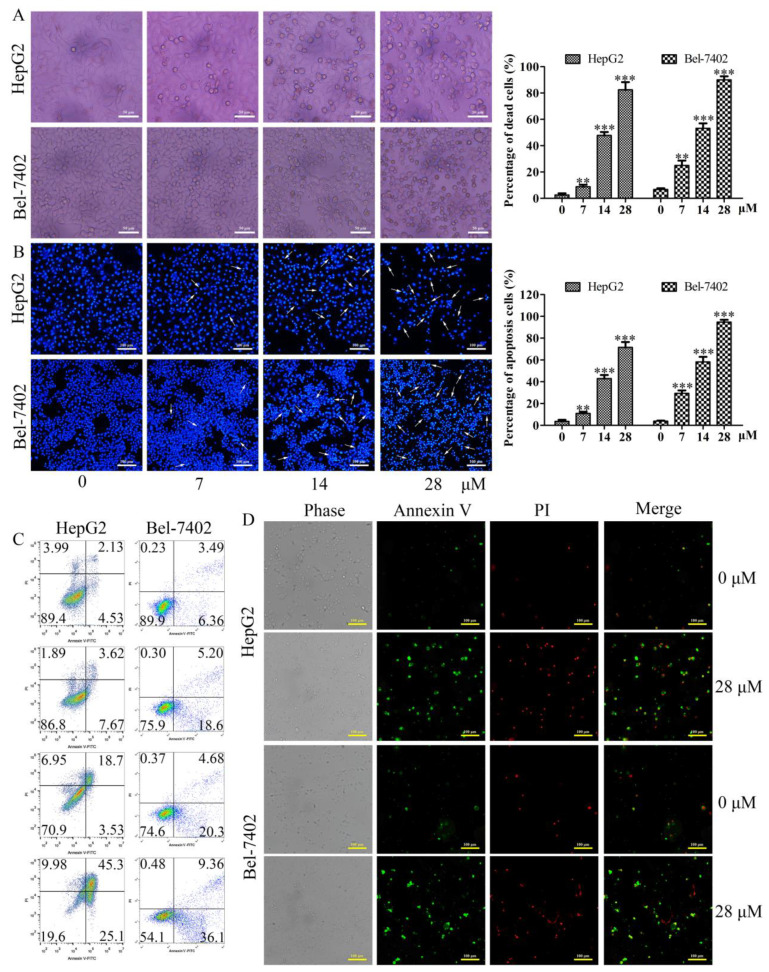
Morphological changes and apoptosis changes of HepG2 and Bel-7402 cells were treated with Davidone C. (**A**,**B**) Hoechst 33258 was used to photograph the morphology of HepG2 and Bel-7402 cells under a fluorescence microscope (80×); (**C**,**D**) HepG2 and Bel-7402 cells were treated with Davidone C for 12 h, then stained with Annexin V/PI, then evaluated by flow cytometry, and photographed under a fluorescence microscope (80×) (** *p* < 0.01, *** *p* < 0.001).

The morphological changes of HepG2 and Bel-7402 cells treated with different concentrations of davidone C were observed under an inverted phase contrast microscope. It was observed that with the increase of the dose, the adherent cells gradually decreased, and the floating cells gradually increased. At high doses, the cells were almost completely shed (Figure 3A).

After staining (Figure 3B), compared with the drug group, the blank group had complete cells and uniform staining. Apoptosis, chromatin agglutination, fragmentation, and bright apoptotic bodies appeared in the drug group, especially in the high-dose group.

To check whether davidone C can induce apoptosis in HepG2 and Bel-7402 cells after 12 h, the cells were stained with Annexin V-FITC and analyzed by flow cytometry. The experimental data takes Annexin V as the horizontal axis and PI as the vertical axis. The upper left quadrant is mechanically damaged cells, the upper right quadrant is late apoptotic or necrotic cells, the lower left quadrant is negative normal cells, and the lower right quadrant is early apoptotic cells. Compared with the control group, the percentage of apoptosis of HepG2 cells was significantly increased after davidone C at different concentrations (0, 7, 14, 28 µM) was administered (Figure 3C). After being treated with different concentrations (0, 7, 14, 28 µM) of davidone C, the percentage of apoptosis of Bel-7402 cells was also significantly higher than that of the control group (Figure 3C). And the experimental results were photographed and observed under the microscope (Figure 3D).

### 2.4. Effects of Davidone C on Mitochondrial Apoptotic Pathway in HepG2 and Bel-7402 Cells

**Figure 4:**
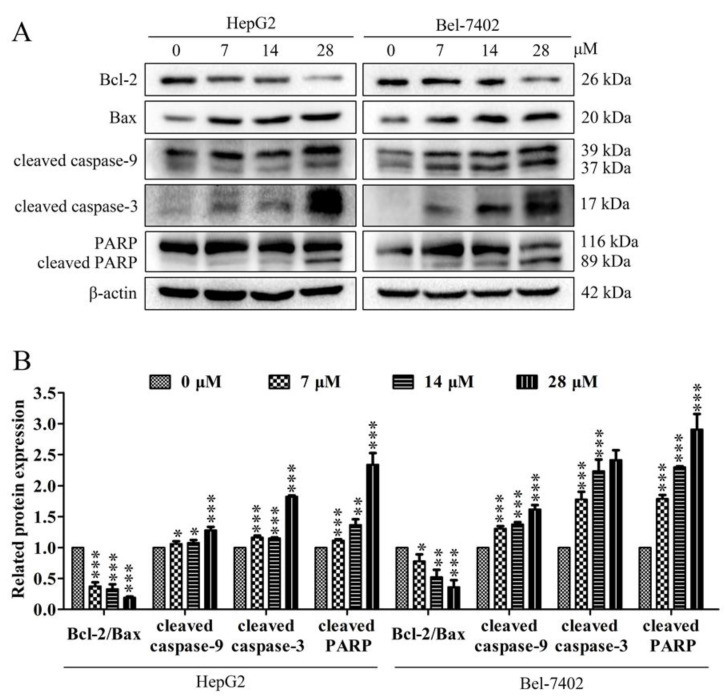
Effects of Davidone C on the apoptosis regulatory proteins of liver cancer cells. (**A**,**B**) The related expressions of Bcl-2/Bax, cleaved caspase-9, cleaved caspase-3 and cleaved PARP in HepG2 and Bel-7402cells were detected by Western blotting. β-actin was used as a control (* *p* < 0.05, ** *p* < 0.01 or *** *p* < 0.001).

In the process of cell apoptosis, the expression of Bcl-2 protein of the Bcl-2 family in mitochondria is down-regulated, and the expression of Bax is up-regulated, which will cause the cytochrome c in the mitochondria to be released into the cytoplasm. At the same time, caspase also plays an important role in the apoptosis signaling pathway network. The pathway of apoptosis depends on the activation of caspase, and the cleavage and activation of caspase is a key step in the final execution of apoptosis. To determine the mechanism of davidone C inducing apoptosis in HepG2 and Bel-7402 cells, we used Western Blot analysis to verify whether davidone C activates mitochondrial apoptosis-related proteins, including the expression of Bcl-2, Bax, cleaved caspase-9, cleaved caspase-3 and cleaved PARP. Treatment with different concentrations (0, 7, 14, 28 µM) of davidone C in HepG2 and Bel-7402 cells increased the expression of Bax and decreased the expression of Bcl-2 (Figure 4). In addition, davidone C increased the expression of cleaved caspase-3, caspase-9 and cleaved PARP (Figure 4).

### 2.5. Effect of Davidone C on Endoplasmic Reticulum Stress (ERS) Pathway in HepG2 and Bel-7402 Cells

**Figure 5:**
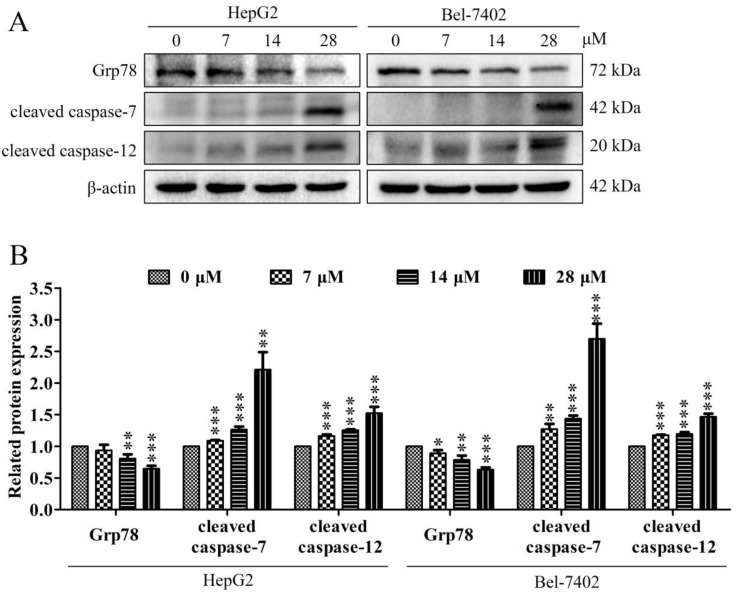
Effects of davidone C on the ERS related proteins of liver cancer cells. (**A**, **B**) The related expressions of Grp78, caspase-7 and caspase-12 in HepG2 and Bel-7402 cells were detected by Western blotting. β-actin was used as a control (* *p* < 0.05, ** *p* < 0.01 or *** *p* < 0.001).

The ERS pathway is one of the key ways that can trigger cell apoptosis. The increase in ERS was a potential activation mechanism of cell apoptosis. This is mainly related to the regulation of some specific transcription-related factors, such as Grp78, caspase-7 and caspase-12. The regulation of apoptosis by these proteins is closely related to the upregulation of ERS. Our results show that davidone C administration can down-regulate the expression of Grp78, and simultaneously up-regulate the expression of caspase-7 and caspase-12 (Figure 5).

### 2.6. Effect of Davidone C on Autophagy Pathway in HepG2 and Bel-7402 Cells

**Figure 6:**
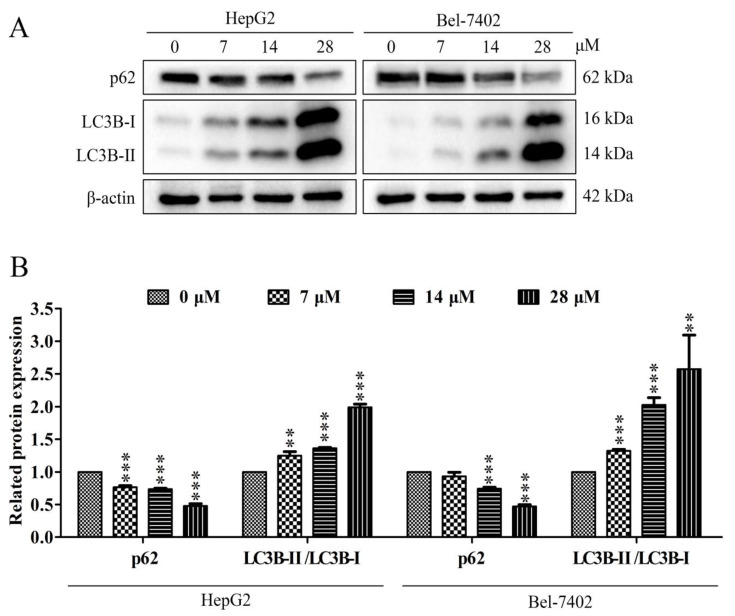
Effects of davidone C on the autophagy related proteins of liver cancer cells. (**A**, **B**) The related expressions of p62, LC3-I and LC3-II in HepG2 and Bel-7402 cells were detected by Western blotting. β-actin was used as a control (* *p* < 0.05, ** *p* < 0.01 or *** *p* < 0.001).

Autophagy can be a survival mechanism and it can also induce cell death. LC3 is a specific protein in the early stage of autophagy. During the process of autophagy, LC3-I is converted to LC3-II. Therefore, LC3 is a key marker protein for autophagy in cells. And p62 is a regulatory protein in the process of autophagy, which can inhibit autophagy. Our results showed that davidone C administration can down-regulate the expression of p62, and simultaneously up-regulate the expression of LC3-I and LC3-II (Figure 6).

### 2.7. Effect of Chloroquine on Autophagy and Apoptosis Pathway in HepG2 and Bel-7402 Cells

**Figure 7:**
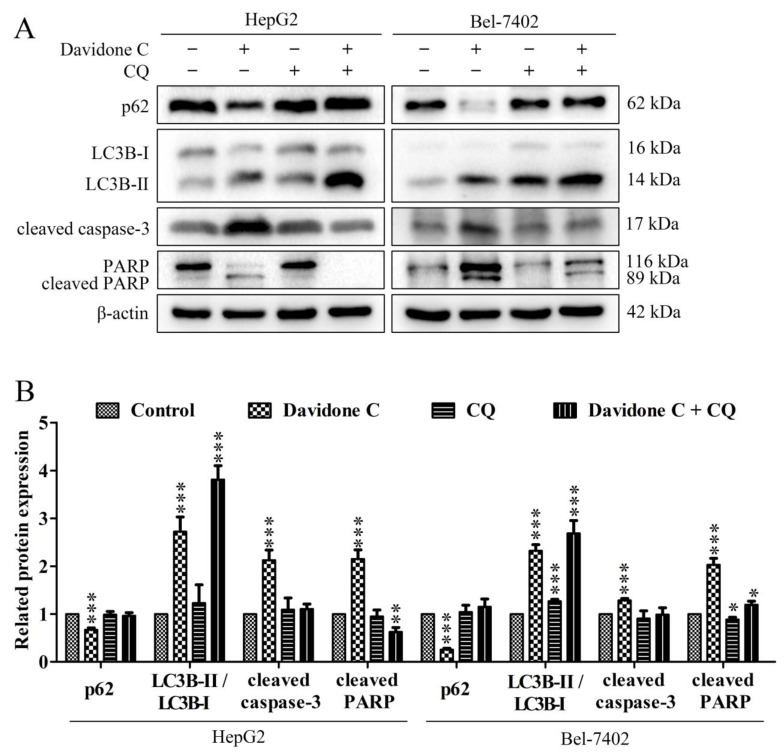
Effects of davidone C with chloroquine on the related proteins of autophagy and apoptosis pathways in HepG2 and Bel-7402 cancer cells. (**A**, **B**) The related expressions of p62, LC3-II, cleaved caspase-3 and cleaved PARP in HepG2 and Bel-7402 cells were detected by Western blotting. β-actin was used as a control (* *p* < 0.05, ** *p* < 0.01 or *** *p* < 0.001).

The use of autophagy inhibitor chloroquine (CQ) can inhibit autophagy before autophagosome formation. As shown in Figure 7, CQ treatment significantly reduced the autophagy and apoptosis induced by davidone C. Compared with the davidone C treatment alone, the CQ pretreatment group significantly increased the expression of p62 and LC3-II, while the expression of cleaved caspase-3 and PARP decreased significantly, which indicated the process of cell autophagy and apoptosis was suppressed. In other words, davidone C induced autophagy in liver cancer cells. When autophagy was inhibited, it further inhibited cell apoptosis, indicating that autophagy might occur upstream of apoptosis.

## 3. Discussion

Previous studies have shown that the ethyl acetate-soluble fraction of *Sophora davidii* (Franch.) Skeels has a certain cytotoxic effect on several human cancer cell lines [15]. However, the anti-tumor activity of specific compounds at the ethyl acetate site has not been reported. In this article, we found that davidone C inhibited the growth of human hepatocellular carcinoma HepG2 and Bel-7402 cells in a time- and concentration-dependent manner (Figure 1). In the cell scratch experiment (Figure 2), compared with the control group, the wound surface area of the davidone C-treated group has a poorer recovery ability in time and concentration dependence. At the same time, morphological observation (picture3) also proved that davidone C can inhibit the growth of hepatocellular carcinoma cells HepG2 and Bel-7402 in a concentration-dependent manner. The results showed that davidone C has significant anti-tumor activity.

There are two different ways of cell death: apoptosis and cell death caused by autophagy transition [16]. Apoptosis belongs to programmed cell death. The main purpose of apoptosis is to remove damaged cells. The disorder of apoptosis mechanism is related to the pathogenesis of cancer [17,18,19]. At present, most clinical anti-cancer drug therapies exert their efficacy through the apoptosis pathway [20]. Autophagy is an evolutionarily conserved important process for the turnover of intracellular substances in eukaryotes. In this process, some damaged proteins or organelles are encapsulated by autophagic vesicles with a double-layer membrane structure, and then sent to lysosomes (animals) or vacuoles (yeasts and plants) for degradation and recycling [21,22]. This study shows that the cell morphology has changed significantly (Figure 3A,B): the cell edges are irregular, the adhesion ability is reduced, the chromatin is condensed, the fluorescence becomes dense, and bright blue dots (apoptotic bodies) are visible. Annexin V-FITC/PI double staining (Figure 3C,D) also confirmed that davidone C can induce cell apoptosis.

Chemotherapeutic drugs induce cell apoptosis by acting on different target proteins in different signaling pathways, such as mitochondrial apoptosis pathway and ERS signaling pathway [23]. The intrinsic apoptotic pathway (mitochondrial dependent) is mediated by intracellular signal molecules, and intracellular signals are transmitted to mitochondria. Subsequently, the pro-apoptotic BH3 members (Bax, Bak) of the Bcl-2 family are activated, and the anti-apoptotic proteins Bcl-2, BCL-XL and Mcl-1 are inhibited, resulting in the destruction of the outer mitochondrial membrane permeability (MOMP) and cytochrome c diffusing into the cytoplasm. Cytochrome c binds to cytoplasmic apoptotic protease activator 1 (Apaf-1) and triggers the formation of a complex called apoptotic bodies. The complex recruits the promoter pro-Caspase-9 to its caspase recruitment domain (CARD) and then undergoes proteolysis. Then, this process activates the downstream executor Caspase-3, lyses cell substrates and induces apoptosis [24,25]. In our research, we explored the possible molecular mechanism of davidone C against hepatocellular carcinoma. Western blotting was used to detect the expression of 5 related proteins (capase-9, caspase-3, cleaved PARP, Bcl-2 and Bax) in HepG2 and Bel-7402 cells treated with different concentrations of davidone C. The current results showed that treatment with Davidone C can increase the expression of caspase-3, caspase-9, clesved PARP and Bax and decrease the expression of Bcl-2 (Figure 4).

The ERS pathway is one of the key ways that can trigger cell apoptosis [26]. Various toxic injuries may cause ERS, and eventually lead to cell apoptosis. The increase of ERS **is** a potential activation mechanism of apoptosis, which is mainly related to the regulation of some specific transcription-related factors, such as the expression of CHOP and caspase-12. The regulation of apoptosis by these proteins is closely related to the upregulation of ERS. Current studies have shown that these molecules can be used as ERS marker proteins, and through the regulation of the expression of related proteins, they can effectively regulate pathways such as apoptosis and cell cycle [27,28]. Studies have shown that the effective compound in American ginseng can regulate cell apoptosis through ERS and oxidative stress [29]. GRP78 is a regulator of ERS, which can protect cells from apoptosis in a variety of ways. It has been reported that caspase activation is required for ERS-mediated cell death. When cells are exposed to ERS, caspase-7 is transported from the cytoplasm to the cytoplasmic surface of the ER membrane, binds to pro-caspase-12 and cleaves pro-caspase-12. After caspase-12 is activated, caspase-3 is activated downstream, leading to cell apoptosis [30]. The results confirmed our conjecture. Davidone C treatment can affect the expression of ERS-related proteins (Figure 5), and indicates that davidone C may promote tumor cell apoptosis through the ERS pathway.

Autophagy is a cell survival pathway controlled by a high degree of signal regulation. It is widely found in the death process of cancer cells. It can be regulated by different signal molecules [31]. The development process of autophagy usually includes initiation, nucleation of autophagosomes, expansion and elongation of autophagosome membranes, closure, and fusion with lysosomes, and finally the degradation of products in vesicles [32]. Start-up begins with the combination of ATG4B and ATG7, and the combination of LC3-I and PE forms LC3-II (also known as MAP1LC3B). Eventually, autophagosomes fuse with lysosomes, the contents are degraded, and macromolecular precursors are recovered or used to provide fuel for metabolic pathways. The adaptor protein sequestosome 1 (also called p62) that targets specific substrates to autophagosomes and LC3-II is degraded together with other cargo proteins and can be used as a measure of autophagic flux [33,34]. After the cells were treated with davidone C, a down-regulation of p62 and an increase in LC3B-I/LC3B-II were observed (Figure 6). This indicated that autophagy is a way of cell death induced by davidone C. At the same time, after adding CQ pretreatment, compared with davidone C alone, the expression of p62 increased, while the expression of cleaved caspase-3 and cleaved PARP decreased (Figure 7). This indicates that the addition of autophagy inhibitors can simultaneously reduce cell autophagy and apoptosis.

## 4. Materials and Methods

### 4.1. Chemicals and Reagents

Chromatography grade solvents were used for HPLC, and all other chemical reagents were analytical grade. HPLC grade acetonitrile and methanol were purchased from Merck (Darmstadt, Germany). Sephadex LH-20 dextran gel was purchased from Amersham Pharmacia Biotech Co., (Piscataway, NJ, USA). The flow double staining kit was purchased from BD Pharmingen (Franklin Lakes, NJ, USA). DMEM medium and fetal bovine serum (FBS), and antibiotics (10 U/mL penicillin G sodium salt and 100 μg/mL streptomycin sulfate) were obtained from Hyclone (Logan, UT, USA).

### 4.2. General Experimental Procedures

Semi-preparative HPLC purification was performed on a Waters 2535 HPLC connected with a 2998 PDA Detector and a 2707 Autosampler (Waters, Milford, MA, USA). Separations were performed on a COSMOSIL C18 column (5 μm, 10 × 150 mm) (Nacalai Tesque, Kyoto, Japan) and a COSMOSIL C8 column (5 μm, 10 × 150 mm) (Nacalai Tesque, Kyoto, Japan). Direct injection high resolution ESIMS and LC-DAD-ESIMS analyses were recorded on an ultra-performance liquid chromatography-quadrupole/electrostatic field orbitrap high resolution mass spectrometry (Thermo Fisher Scientific, Waltham, MA, USA).The NMR spectra were recorded on an AVANCE III 600 MHz spectrometer (Bruker BioSpin, Ettlingen, Germany). Optical rotations were recorded on an Autopol IV Automatic Polarimeter (Rudolph Research Analytical, Hackettstown, NJ, USA).

### 4.3. Source and Isolation of Sophora Davidii (Franch.) Skeels

Roots of *S. davidii* (Franch.) Skeels (age 12–15 years) were collected from Xiuwen county, Guizhou Province, China (at altitudes of 1200 to 1300 m), in June 2014. The roots were dried at room temperature, macerated into a fine powder, and stored at room temperature. The identification was done by Professor Dingrong Wan of the School of Pharmaceutical Sciences, South-Central University for Nationalities (SCUN, Wuhan, China). A voucher specimen (SC0801) is deposited in the SCUN School of Pharmaceutical Sciences.

### 4.4. Extraction and Isolation

The dried roots of the plant (18 kg) were milled and then extracted with 80% EtOH (4 × 20 L, 3 days each) at room temperature to yield 850 g of crude EtOH extract. Subsequently, the EtOH fraction was suspended in H_2_O and partitioned with petroleum ether (PE) (4 × 10 L), ethyl acetate (EtOAc) (4 × 10 L), and *n*-butyl alcohol (*n*-BuOH) (4 × 10 L) to give a PE extract (90 g), EtOAc extract (215 g), and *n*-BuOH extract (110 g), respectively. The EtOAc part (200 g) was subjected to silica-gel column chromatography (300–400 mesh) eluting with a gradient solvent system of CH_2_Cl_2_/MeOH (200:1 to 0:1, *v*/*v*) to yield sixteen fractions (F1–F16). Fraction 13 (8.8 g) was further separated on silica gel (10–40 μm) eluted with CH_2_Cl_2_/MeOH (50:1 to 3:1, *v*/*v*) to obtain 8 fractions (F13-1-F13-8). F13-7 (1.2 g) was further purified by Sephadex-LH20 eluted with MeOH followed by C_18_ reverse phase HPLC respectively to give davidone C (106.6 mg). The purity of davidone C was more than 95% according to the HPLC analysis.

### 4.5. Cell Culture

The human hepatocellular carcinoma cell lines HepG2 and Bel-7402 were purchased from the American Type Culture Collection (ATCC; Manassas, VA, USA). HepG2 and Bel-7402 cells were grown in Dulbecco’s modified Eagle’s medium (DMEM) (Sigma-Aldrich, St. Louis, MO, USA) supplemented with 10% FBS, 1% 100 U/mL of penicillin and 100 μg/mL of streptomycin (Hyclone, Logan, UT, USA). The cells were cultured with 5% CO_2_ at 37 °C.

### 4.6. MTT Assay

The MTT assay was used to evaluate the toxicity of davidone C in HepG2 and Bel-7402 cell lines. The cells in the logarithmic growth phase were seeded in a 96-well plate and cultured to 90% confluence and then treated with davidone C (0, 5, 10, 20, 30 or 40 µM) for 6, 12 or 24 h. Next, 5 mg/mL of MTT was added to each well, and the cells were incubated at 37°C for half an hour. Subsequently, the medium was discarded and dimethyl sulfoxide (DMSO, 150 µL) was added to each well. The absorbance of each well was measured at 562 nm using a microplate reader (Bio-Rad, CA, USA).

The formula used to calculate the cell inhibition rate (%) is as follows:

Cell growth inhibition rate (%) = [(OD control group-OD sample group)/(OD control group-OD blank group)] × 100% [35].

GraphPad Prism 6.0 software (GraphPad Software Inc., San Diego, CA, USA) was used to calculate the IC_50_ values of davidone C.

### 4.7. Scratch Assay

In the cell migration experiment, HepG2 and Bel-7402 cells in the logarithmic growth phase were seeded onto 6-well plate, covering the plate until 90%, and then scraping 6 wells with a sterile 200 µL pipette tip. Next, the cells were treated with 0, 2.5, 5 or 10 µM davidone C for 12 h. The cell morphology was observed, then a fluorescence inverted microscope was used to take pictures of the cells. The ImageJ software was used to analyze the results.

### 4.8. Hoechst 33258 Stain

HepG2 and Bel-7402 cells in logarithmic growth phase were inoculated onto 6-well plate and covered the plates until 90% confluence. Next, the cells were treated with 0, 7, 14 and 28 µM davidone C for 12 h. The supernatant from each well was discarded the cells and fixed with a fixing solution (methanol: acetic acid = 3:1) for 15 min. Then phosphate buffer saline (PBS) was added carefully and the cells aspirated and the fluid discard after washing once. After air-drying, Hoechst 33258 staining solution (5 µg/mL) diluted with PBS was added to each well. After that, the cell morphology was observed and photographed using a fluorescence inverted microscope at 40 × magnification (excitation wavelength at 350 nm, emission wavelength at 460 nm).

### 4.9. Annexin V- FITC Double Staining

HepG2 and Bel-7402 cells in logarithmic growth phase were cultured to 90% confluence in 6-well plates. Next, the cells were treated with davidone C at concentrations of 0, 7, 14 and 28 µM for 12 h. The cells were washed twice with PBS and centrifuged. The cells were then resuspended in 400 µL of 1 × binding buffer, and 5 µL of Annexin V-FITC and 5 µL of PI were added to the cells in the dark and incubated for 10 mi. Finally, flow cytometry was used to analyze the apoptosis rate, and a fluorescent inverted microscope was used to take photos and record at 40 × magnification.

### 4.10. Western Blotting

1 × 10^6^ HepG2 and Bel-7402 cells were seeded in 6-well plates and treated with 0, 7, 14 and 28 µM davidone C for 12 h. Next, the total protein was extracted from the cells and a bicinchoninic acid (BCA) protein kit was used to determine the protein content of each sample. The same amount of protein sample was immobilized by 10% sodium salt for polyacrylamide gel electrophoresis (SDS-PAGE) and transferred to a polyvinylidene fluoride (PVDF) membrane by electrophoresis. The PVDF membrane was then blocked with skimmed milk powder and incubated with the corresponding antibody at 4 °C overnight. The next day, the membrane was washed 3 times with Tris-HCl buffered salt solution with pH 7.4 plus appropriate concentration of Tween (TBST) and incubated with the corresponding secondary antibody at room temperature for 2 h. Finally, the membrane was washed with TBST and finally developed with ECL kit. The changes in protein expression were analyzed using the Image lab software.

### 4.11. Statistical Analysis

The data were expressed as mean ± SD at least three separate experiments. GraphPad Prism 6.0 software was used for all statistical analyses. Statistical differences were analyzed by one-way analysis of variance (ANOVA) and statistical significance was determined as * *p* < 0.05, ** *p* < 0.01 or *** *p* < 0.001.

## 5. Conclusions

Malignant tumors are one of the diseases with the highest morbidity and mortality in the world. The use of chemotherapeutic drugs can effectively inhibit the induction of tumor cell death, thereby inhibiting the proliferation of malignant tumors, and improving the cure rate and survival rate of cancer patients. We proved here that davidone C isolated from *S*. *davidii* can induce tumor cell death by activating mitochondrial apoptosis, endoplasmic reticulum stress and autophagy pathways. Our results can inspire researchers to continue on anti-hepatocellular carcinoma activity and mechanism of davidone C and provide evidence for its development as a potential anti-tumor agent.

## Data Availability

The data presented in this study are available in Supplementary Materials.

## Supplementary Materials

The following are available online. Figures S1–S5: Original images of Western blotting for Figure 2, Figure 4, Figure 5, Figure 6 and Figure 7; Figure S6: HepG2 and Bel-7402 cells were treated with Davidone C, 3-MA, and Davidone C + 3-MA for 24 h and then stained with Annexin V/PI staining and then evaluated by flow cytometry.

## Sample Availability

Samples of davidone C are available from the authors.
