# Supplementary material for: Davidone C Induces the Death of Hepatocellular Carcinoma Cells by Promoting Apoptosis and Autophagy
Source: Molecules. 2021 Aug 28;26(17):5219. doi: 10.3390/molecules26175219 (PMC8434093; doi:10.3390/molecules26175219)
Supplement: Supplementary file 1 [file molecules-26-05219-s001.zip › molecules-1287927-supplementary.pdf]

Supporting Information

Davidone C Induces the Death of Hepatocellular Carcinoma Cells by Promoting Apoptosis and Autophagy

Ping Song [1], Huiqi Huang [2], Yuanren Ma [2], Chaoqun Wu [2], Xinzhou Yang [2,*] and Ho-Young Choi [3,*]

[1] School of Chemistry and Chemical Engineering, Qinghai University for Nationalities, Xining 810007, China; spzhe@126.com
[2] School of Pharmaceutical Sciences, South-Central University for Nationalities, Wuhan 430074, China; Hhuiqi@hotmail.com (H.H.); 13007136998@163.com (Y.M.); wcqscuec@126.com (C.W.)
[3] College of Korean Medicine, Kyung Hee University, Seoul 02447, Republic of Korea
* Correspondence: xzyang@mail.scuec.edu.cn, (X.Y.) Tel.: +86-27-6784-1196 (X.Y.); hychoi@khu.ac.kr (H.-Y.C.); Tel.: +82-2-9619372 (H.-Y.C.)

CONTENT

Figure S1 Original images of Western blotting (Figure 2)
Figure S2 Original images of Western blotting (Figure 4)
Figure S3 Original images of Western blotting (Figure 5)
Figure S4 Original images of Western blotting (Figure 6)
Figure S5 Original images of Western blotting (Figure 7)
Figure S6 HepG2 and Bel-7402 cells were treated with Davidone C, 3-MA, and Davidone C + 3-MA for 24 h and then stained with Annexin V/PI staining and then evaluated by flow cytometry.

Figure 2

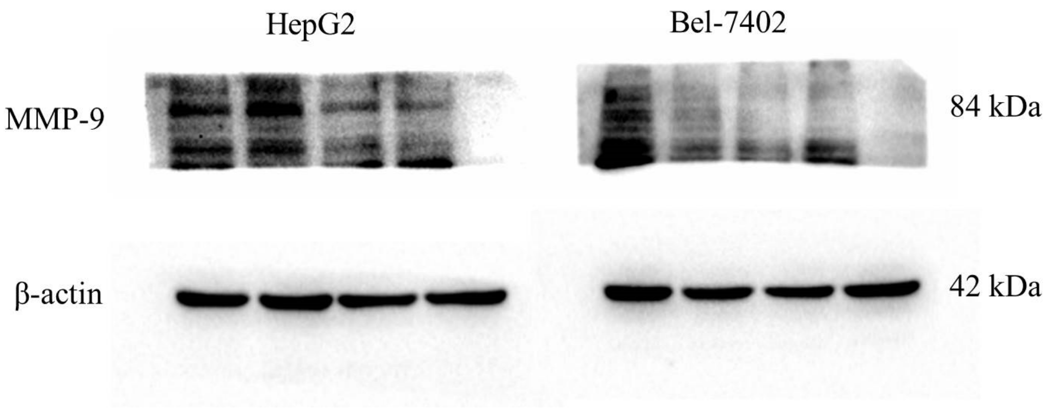

Figure S1. Original images of Western blotting (Figure 2).

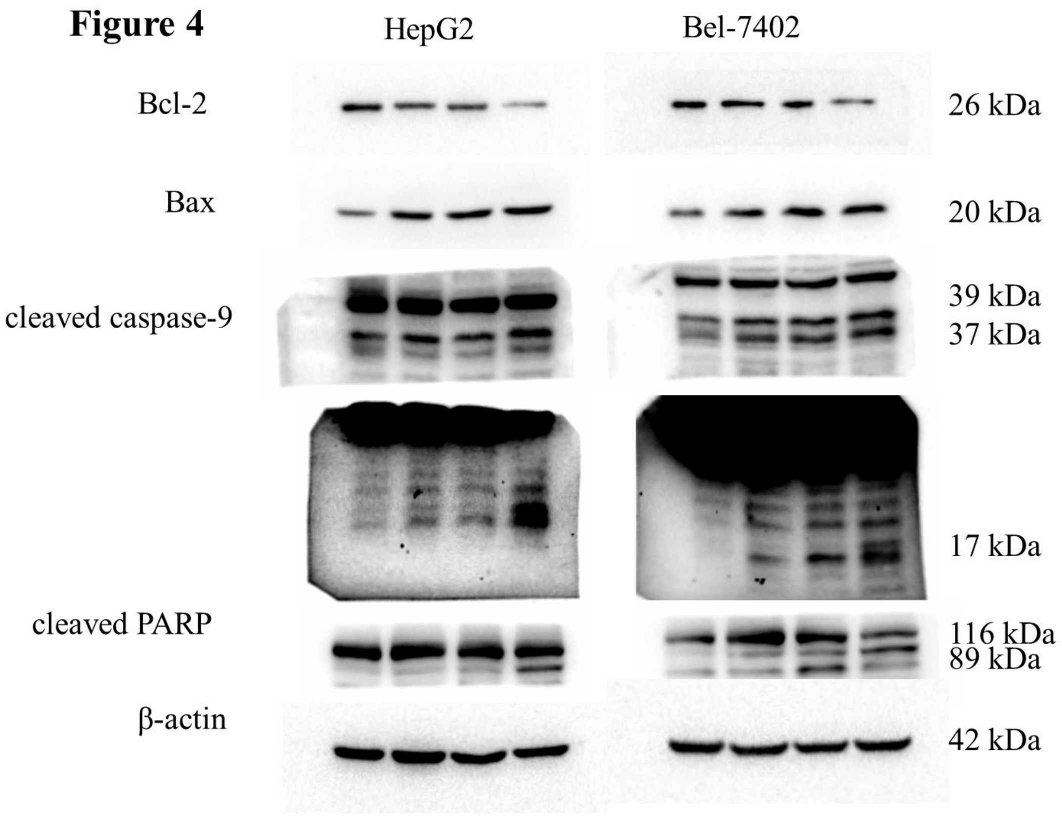

Figure S2. Original images of Western blotting (Figure 4).

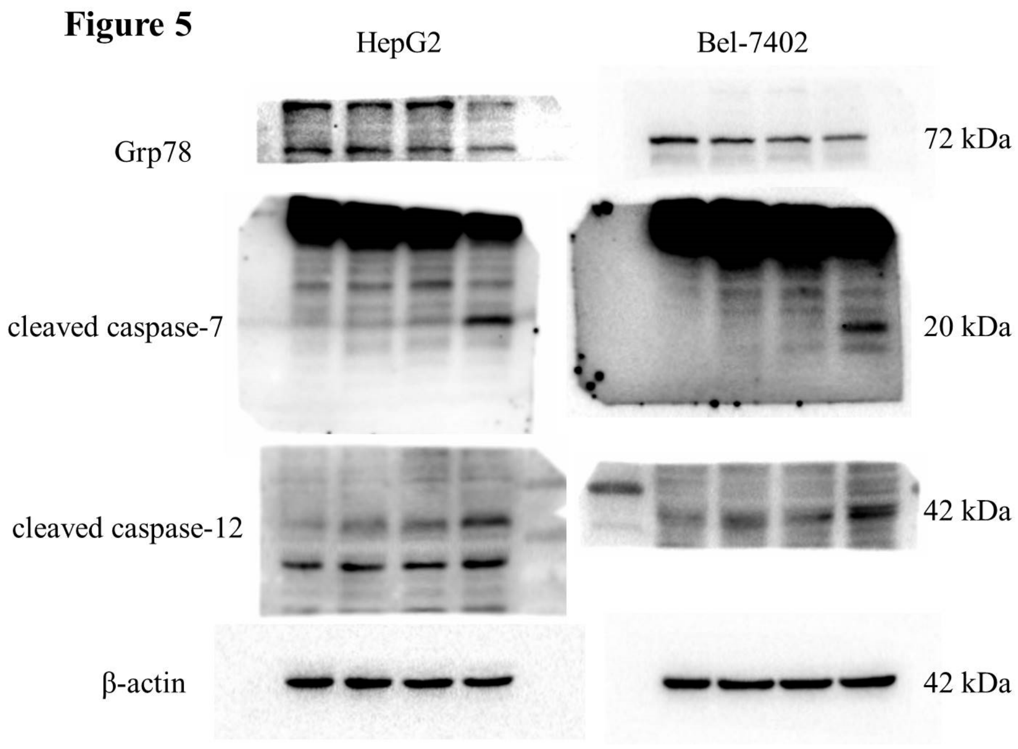

Figure S3. Original images of Western blotting (Figure 5).

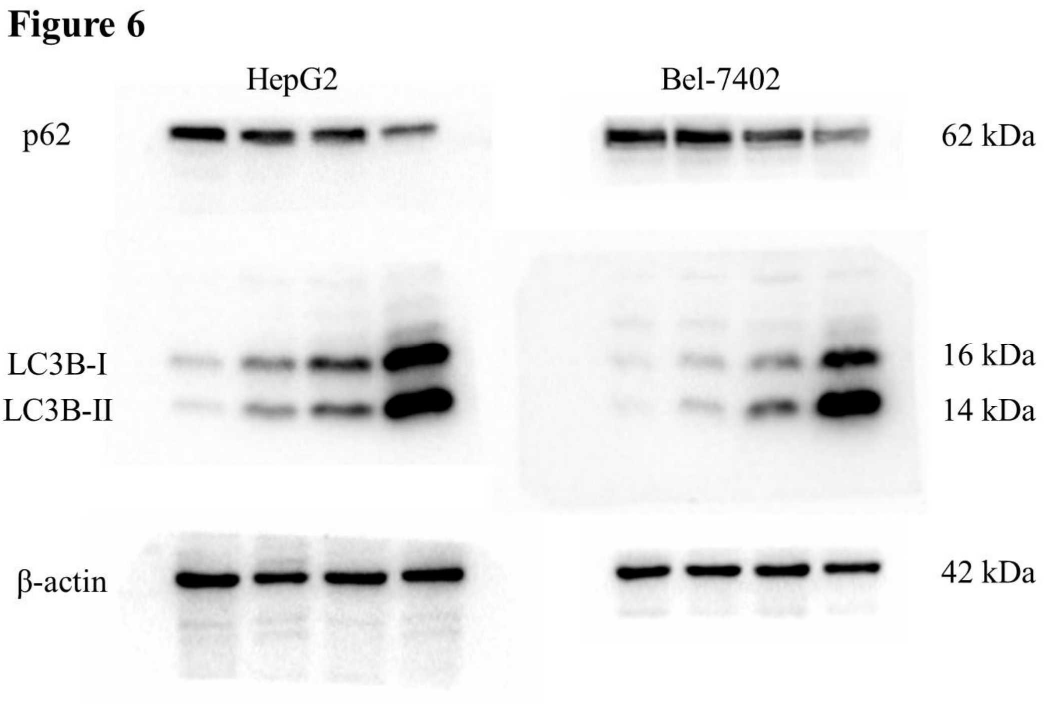

Figure S4. Original images of Western blotting (Figure 6).

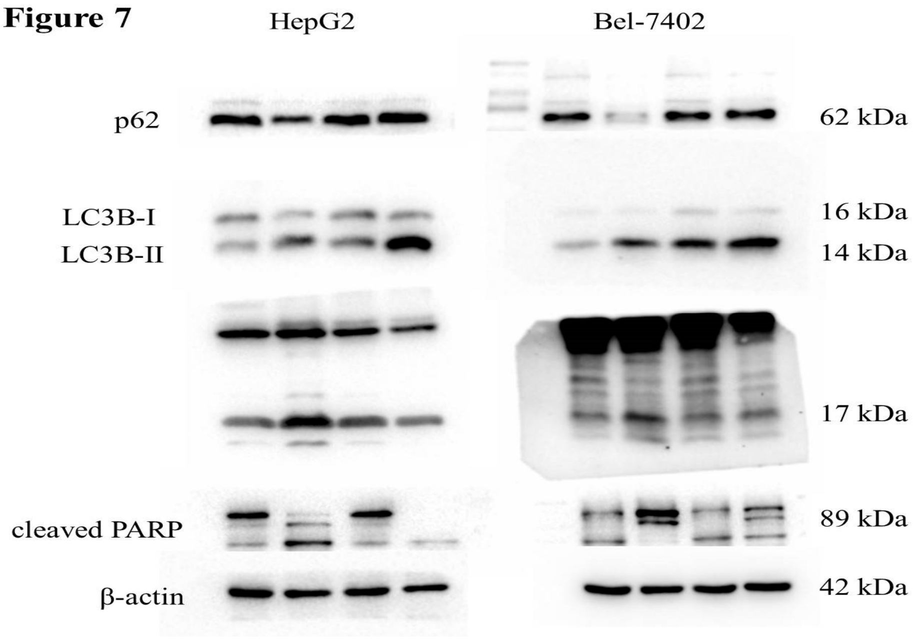

Figure S5. Original images of Western blotting (Figure 7).

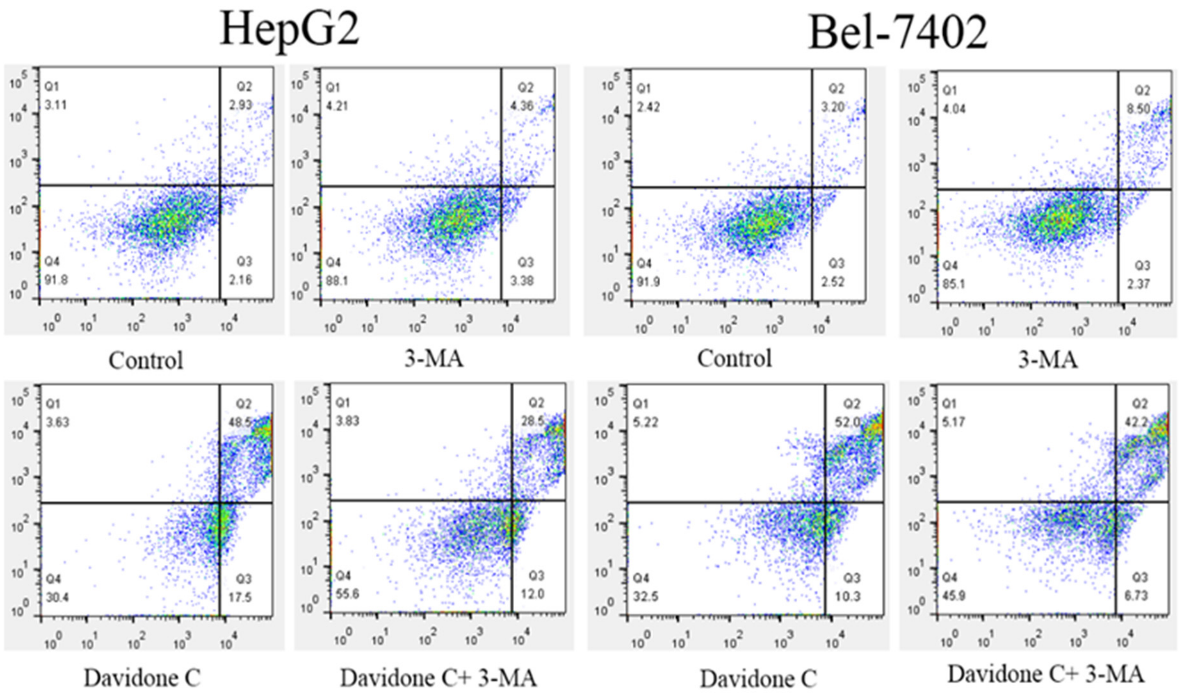

Figure S6. HepG2 and Bel-7402 cells were treated with Davidone C, 3-MA, and Davidone C + 3-MA for 24 h and then stained with Annexin V/PI staining and then evaluated by flow cytometry.
